# Three new minute leaf litter beetles discovered by citizen scientists in Maliau Basin, Malaysian Borneo (Coleoptera: Leiodidae, Chrysomelidae)

**DOI:** 10.3897/BDJ.5.e21947

**Published:** 2017-12-04

**Authors:** Menno Schilthuizen, Lilian A Seip, Sean Otani, Jadda Suhaimi, Iva Njunjić

**Affiliations:** 1 Taxon Expeditions, Leiden, Netherlands; 2 Naturalis Biodiversity Center, Leiden, Netherlands; 3 Universiti Malaysia Sabah, Kota Kinabalu, Malaysia; 4 Tottori University, Tottori, Japan; 5 Tottori University of Environmental Studies, Tottori, Japan; 6 Yayasan Sabah, Kota Kinabalu, Malaysia

**Keywords:** Leiodinae, Galerucinae, Southeast Asia, tropical rainforest, new species, taxon expeditions

## Abstract

**Background:**

We coin the term "taxon expeditions" for citizen scientists' field courses to carry out publishable taxonomic work in close association with trained taxonomists.

**New information:**

During the first-ever taxon expedition, in Maliau Basin Studies Centre, Sabah, Malaysian Borneo, the participants sampled leaf litter beetles from lowland dipterocarp forest using the Winkler apparatus. The collected material proved to contain at least three undescribed species of small-bodied (ca. 1 mm long) hemispherical litter-dwelling Coleoptera. As part of the field course work, taxonomic descriptions were prepared for the chrysomelid *Clavicornaltica sabahensis* sp. n. and the leiodids *Colenisia chungi* sp. n. and *Dermatohomoeus maliauensis* sp. n.

## Introduction

During tropical biology field courses, it is common for students to practise field ecological methods using quantified sampling of various groups of invertebrates. Except for rare exceptions (e.g. Miller et al. 2014), after sorting and (coarse) identification, the specimens are usually discarded or stored as bulk samples, even though the materials are likely to be of taxonomic importance. We have recently begun a series of field courses for citizen scientists (which we term "taxon expeditions"; see http://www.taxonexpeditions.com) in which a taxonomic treatment of selected taxa forms a central part of the course work. We believe that this serves two important purposes: (i) a better appreciation for the practice of taxonomy amongst the general public and (ii) valid taxonomic output.

The Taxon Expeditions concept involves ten days of lectures and workshops in a well-equipped field research centre, during which the citizen scientists are trained in basic field and lab. techniques for biodiversity assessment and identification. Under the guidance of embedded taxonomists, the participants identify and describe new taxa belonging to the taxonomists' field of expertise. The collected materials are stored in a local collection and provided with voucher numbers that are referred to in all output such as published taxonomic treatments, web-based taxonomy platforms, and 3rd-generation DNA sequencing of DNA barcodes in the field (Menegon et al. 2017).

In this paper, we provide the first output from this model, viz. taxonomic treatments for three new species of minute Coleoptera that were collected from leaf litter in tropical lowland forests in Borneo using Winkler extraction. The taxa belong to (a) the leaf beetle genus *Clavicornaltica* Scherer, 1974 (a relatively recently discovered genus that is likely to be highly diverse in tropical leaf litter biotas; Scherer (1974), Konstantinov and Duckett (2005)) and (b) the leiodid genera *Dermatohomoeus* Hlisnikovský, 1963 and *Colenisia* Fauvel, 1903, two speciose genera that are widespread thoughout Africa, Asia, Australia, and the Pacific islands, but which have not yet been studied extensively in Borneo. An accompanying paper on Elmidae (Coleoptera) will appear elsewhere.

## Materials and methods

We sampled at 4.7389°N, 116.9696°E, at 260 m elevation, at a location where a small stream enters the Maliau River along the Seraya Trail of the Maliau Basin Studies Centre (Sabah, Malaysian Borneo). Six course participants and two course instructors collected leaf litter from the space between the plank roots of large trees ("buttress sample") and from the open forest floor in between ("floor sample"). The two samples were similar in amount (ca. 15 l). The litter was first sieved using a 1-cm-mesh beetle sieve (Fig. 1) and the flow-through was then placed inside Winkler bags, which accumulated the emerging invertebrates in pure ethanol over a period of four days. Then, the Coleoptera were picked from the samples and from these, the Leiodinae (Leiodidae) and Galerucinae (Chrysomelidae) were selected for further treatment.

In the buttress sample, 1 specimen of *Clavicornaltica* (Galerucinae), 2 specimens of *Colenisia* Fauvel and 2 specimens of *Dermatohomoeus* Hlisnikovský (Leiodinae: Pseudoliodini) were found. The floor sample yielded no materials from these genera.

The specimens were studied and their morphological features documented using the limited equipment available at the field centre, i.e. a Nikon SMZ445 with 20x eye pieces (magnification up to 70x), a Canon EOS 500D with MP-E 65 mm lens placed on a Kaiser copy stand with micro-drive, and basic dissection materials. Lengths of body parts were measured by photographing a ruler with 0.5 mm line spacing alongside the specimen and then measuring both the ruler and the body parts from the photographs. Ratios of antennomeres and body parts were also calculated from measurements taken from photographs. Spacing of punctures and other microsculptural elements was, where possible, measured from electron micrographs. Drawings were done freehand and proportions may therefore deviate somewhat from reality. The material was compared with all relevant taxonomic literature (see below). Dissected genitalia, antennae, and other body parts were embedded in PVP embedding medium (Lompe 1986) and mounted either on a mounting board or on a glass micro-slide and attached to the same pin as the specimen. All specimens were given collection numbers of the Borneensis (BORN) collection of the Institute for Tropical Biology and Conservation at Universiti Malaysia Sabah and stored there permanently.

In addition, specimens were studied of the same genera collected by Winkler extraction at another site in Maliau Basin (Ginseng Camp, 670 m elevation; Chung et al. 2010) and held in the collection of the Forest Research Centre at Sepilok, Sabah (FRCS). These specimens were used for scanning electron micrographs using a JEOL JSM-7600F. The FRCS does not use collection numbers.

## Taxon treatments

### Colenisia

Fauvel, 1903; Type species: Colenisia caledonica Fauvel, 1903

Colenisia Fauvel, 1903; (Fauvel 1903, Newton 1998, Daffner 1991, Švec 2013)
Colenisia Colenisia caledonica Fauvel, 1903Fauvel 1903

#### Diagnosis

Body small (0.8-2.5 mm), oval, glossy, usually uniformly brown. Head broad, occipital crest absent, at least half as wide as the pronotum, with distinct microreticulation. Antennal insertion concealed, antennal groove absent. Antennae 11-segmented, relatively compact, 8th antennomere much smaller than 9th and 10th. Labrum not emarginate; mandible with strongly developed molar surface. Elytra with transverse microreticulation. First abdominal segment without a transverse carina. Hind coxae not separated. Tarsal formula 5-4-4 in both sexes. Aedeagus with free parameres.

### Colenisia chungi

Schilthuizen, Seip & Otani sp. n.

0DEB6D81-4651-4E03-BB41-DE87DE659851

#### Materials

- **Type status:** Holotype. **Occurrence:** catalogNumber: BOR/COL/14090; recordedBy: I. Njunjić; M. Schilthuizen; Taxon Expeditions; individualCount: 1; sex: male; lifeStage: adult; preparations: card-mounted; disposition: in collection; **Taxon:** scientificName: Colenisia chungi; order: Coleoptera; family: Leiodidae; genus: Colenisia; specificEpithet: chungi; taxonRank: species; scientificNameAuthorship: Schilthuizen, Seip & Otani; **Location:** continent: Asia; island: Borneo; country: Malaysia; stateProvince: Sabah; municipality: Tongod; verbatimLocality: Maliau Basin, near Studies Centre, along Seraya Trail, where stream enters the river; verbatimElevation: 260 m; verbatimCoordinateSystem: decimal degrees; decimalLatitude: 4.7389; decimalLongitude: 116.9696; **Event:** samplingProtocol: Winkler, litter from basis of trees; samplingEffort: 15 l of leaf litter; eventDate: 2017-09-27; habitat: lowland dipterocarp forest; fieldNumber: TxEx-MBSC1wb; **Record Level:** institutionID: Universiti Malaysia Sabah; collectionID: Institute for Tropical Biology and Conservation, BORNEENSIS; institutionCode: UMS; collectionCode: BORN; basisOfRecord: PreservedSpecimen
- **Type status:** Paratype. **Occurrence:** catalogNumber: BOR/COL/14091; recordedBy: I. Njunjić; M. Schilthuizen; Taxon Expeditions; individualCount: 1; sex: female; lifeStage: adult; preparations: card-mounted; disposition: in collection; **Taxon:** scientificName: Colenisia chungi; order: Coleoptera; family: Leiodidae; genus: Colenisia; specificEpithet: chungi; taxonRank: species; scientificNameAuthorship: Schilthuizen, Seip & Otani; **Location:** continent: Asia; island: Borneo; country: Malaysia; stateProvince: Sabah; municipality: Tongod; verbatimLocality: Maliau Basin, near Studies Centre, along Seraya Trail, where stream enters the river; verbatimElevation: 260 m; verbatimCoordinateSystem: decimal degrees; decimalLatitude: 4.7389; decimalLongitude: 116.9696; **Event:** samplingProtocol: Winkler, litter from basis of trees; samplingEffort: 15 l of leaf litter; eventDate: 2017-09-27; habitat: lowland dipterocarp forest; fieldNumber: TxEx-MBSC1wb; **Record Level:** institutionID: Universiti Malaysia Sabah; collectionID: Institute for Tropical Biology and Conservation, BORNEENSIS; institutionCode: UMS; collectionCode: BORN; basisOfRecord: PreservedSpecimen
- **Type status:** Paratype. **Occurrence:** recordedBy: A.Y.C. Chung; Momin Binto; J.L. Yukang; individualCount: 1; sex: male; lifeStage: adult; preparations: card-mounted; dissected; coated for SEM; disposition: in collection; **Taxon:** scientificName: Colenisia chungi; order: Coleoptera; family: Leiodidae; genus: Colenisia; specificEpithet: chungi; taxonRank: species; scientificNameAuthorship: Schilthuizen, Seip & Otani; **Location:** continent: Asia; island: Borneo; country: Malaysia; stateProvince: Sabah; municipality: Tongod; verbatimLocality: Maliau Basin, along the Seraya Trail and Agathis Trail near the Ginseng Camp; verbatimElevation: 670 m; verbatimCoordinateSystem: decimal degrees; **Event:** samplingProtocol: Winkler extraction of leaf litter; samplingEffort: 20 1-square-m units of leaf litter and soil; eventDate: 2005-03-06/12; fieldNumber: A1 (3); **Record Level:** institutionID: Forest Research Centre; institutionCode: FRCS; basisOfRecord: PreservedSpecimen

#### Description

Length of body 1.25 mm. Maximum width of elytra 0.86 mm. Head width (including the eyes) 0.46 mm. Greatest width of pronotum 0.8 mm. Winged. Short and oval, shiny and sparsely pubescent, dark chestnut, angles of pronotum, strip along suture, and head dark ochre (Fig. 2). Legs and antennomeres I-VI yellow, antennomeres VII-XI orange. Entire dorsum transversely microsculptured.

**Head**: Ratio of horizontal width of eye (measured in dorsal view and perpendicular to the longitudinal axis of the head) to distance between eyes: 1:7.4. Transverse microsculpture recognisable but too fine to distinguish individual cells at 50x magnification (distance between individual striae is 3-5 µm; Fig. 3a). At 50x magnification, no punctuation is clearly visible. Length of antennomere III 0.8 times the length of antennomere II. Antennomere XI slightly wider than antennomere X (Fig. 5a). Mandible with 16 parallel rows transversely situated on the dorsal molar surface (Fig. 5b).

**Pronotum**: Broadest at the base. Base completely straight to posterior angles. Posterior angles form a right angle, while the tip of the angle itself is slightly rounded. From posterior angles to anterior angles, the pronotum is gently curved inwards. The sides and the anterior angle have a fine groove along the entire margin. Transverse microsculpture slightly less distinct than on the head, individual grooves narrowly spaced (3-5 µm apart), but just visible at 50x magnification (Fig. 3b). Punctures (when viewed at 50x magnification) fine and sparse, separated by 5-10 times their own diameter, bearing short, inconspicuous hairs.

**Scutellum**: Microsculptured as on pronotum.

**Elytra**: Broadest at basal quarter, roundly curved to apex. Surface with transverse microsculpture. Microsculpture much more pronounced than on the pronotum, already recognisable at 15x magnification. Individual horizontal striae separated from one another by ca. 20 µm (about the width of antennomere III). Punctures separated by around 5-8 times their own diameter, irregularly arranged, each with a hair that can be up to 30 µm long (Fig. 3c). Sutural stria absent.

**Legs**: Anterior tarsomeres I-IV not markedly widened in the male.

**Aedeagus**: Median lobe elongated, parallel-sided, at the tip extended into a flat processus reminiscent of a duck-bill. Parameres thin, short, two-thirds of the length of the median lobe, slightly widened at the tip and provided with two long hairs, each about one-third of the length of the paramere itself (Fig. 4a). In lateral view, the median lobe is gently curved and apically flattened into a wedge (Fig. 4b).

**Spermatheca**: A near-spherical bulb with a tube about twice as long as the diameter of the bulb and about a third of its diameter. Tube from its base narrowing to about half its own diameter towards the terminus.

#### Diagnosis

The eye size, dorsal microsculpture, shape of aedeagus and antenna, as well as the presence of irregularly arranged punctuation on the elytra, place this species near *C. championi* (Portevin 1937) from South India, *C. pecki* Daffner 1988 from Japan and *C. castanea* Švec 2011 from China. However, *C. championi* has longer parameres and a less clearly sinuous outline of the aedeagus apex (Daffner 1991). *C. pecki* has the 11th antennomere much smaller than in *C. chungi*. *C. castanea* has acute posterior angles of the pronotum and a more stocky aedeagus (Švec 2011).

#### Etymology

Named in honour of Dr. Arthur Y. C. Chung, who collected the first known specimen in 2005.

#### Distribution

Known only from two locations in the valley where the Maliau river flows out of Maliau Basin, located at 290 m elevation (Maliau Basin Studies Centre) and 670 m elevation (Ginseng Camp).

#### Ecology

Only collected from leaf litter on the forest floor in lowland dipterocarp tropical rainforest. The two specimens from the Maliau Basin Studies Centre were both collected from between buttress roots, whereas leaf litter from the forest floor yielded no specimens. Perhaps this is an indication of its preferred microhabitat.

### Dermatohomoeus

Hlisnikovský, 1963

Dermatohomoeus Hlisnikovský, 1963 (Hlisnikovský 1963, Daffner 1986, Newton 1998)
Dermatohomoeus Dermatohomoeus guineensis Hlisnikovský, 1963Hlisnikovský 1963

#### Diagnosis

Body convex, shiny, brown. Head without any microreticulation but with punctate microsculpture, occipital crest absent, antennal insertion concealed, antennal groove absent, labrum not emarginate. Antenna slender, 11-segmented, 8th antennomere much smaller than 9th and 10th. Mandible with clear molar surface. Pronotum more than twice as wide as the head, with fine and densely placed punctures. Elytra with densely placed punctures and transverse microsculpture. Mesosternum with longitudinal carina. First abdominal sternite without a transverse carina. Hind coxae not separated. Tarsal formula 5-4-4 in males and females. Aedeagus with free parameres.

### Dermatohomoeus maliauensis

Schilthuizen, Otani & Seip sp. n.

D4100AF1-47D2-4D7F-8F90-B8BD6D29E189

#### Materials

- **Type status:** Holotype. **Occurrence:** recordedBy: A.Y.C. Chung; Momim Binti; J.L. Yukang; individualCount: 1; sex: male; lifeStage: adult; preparations: card-mounted; head, legs and genitalia dissected; disposition: in collection; **Taxon:** scientificName: Dermatohomoeus maliauensis; order: Coleoptera; family: Leiodidae; genus: Dermatohomoeus; specificEpithet: maliauensis; taxonRank: species; scientificNameAuthorship: Schilthuizen, Otani & Seip; **Location:** continent: Asia; island: Borneo; country: Malaysia; stateProvince: Sabah; municipality: Tongod; locality: Maliau Basin, along the Seraya Trail and the Agathis Trail near the Ginseng Camp; verbatimElevation: 670 m; **Event:** samplingProtocol: Winkler extraction of leaf litter; samplingEffort: 20 1-square-m units of leaf litter and soil; eventDate: 2005-03-06/12; fieldNumber: S2; **Record Level:** institutionID: Forest Research Centre; institutionCode: FRCS; basisOfRecord: PreservedSpecimen
- **Type status:** Paratype. **Occurrence:** recordedBy: A.Y.C. Chung; Momin Binti; J.L. Yukang; individualCount: 1; sex: male; lifeStage: adult; preparations: card-mounted; head, legs, and genitalia dissected; coated for SEM; disposition: in collection; **Taxon:** scientificName: Dermatohomoeus maliauensis; order: Coleoptera; family: Leiodidae; genus: Dermatohomoeus; specificEpithet: maliauensis; taxonRank: species; scientificNameAuthorship: Schilthuizen, Otani & Seip; **Location:** continent: Asia; island: Borneo; country: Malaysia; stateProvince: Sabah; municipality: Tongod; locality: Maliau Basin, along the Seraya Trail and the Agathis Trail near the Ginseng Camp; verbatimElevation: 670 m; **Event:** samplingProtocol: Winkler extraction of leaf litter; samplingEffort: 20 1-square-m units of leaf litter and soil; eventDate: 2005-03-06/12; fieldNumber: A9; **Record Level:** institutionID: Forest Research Centre; institutionCode: FRCS; basisOfRecord: PreservedSpecimen
- **Type status:** Paratype. **Occurrence:** catalogNumber: BOR/COL/14092; recordedBy: I. Njunjić; M. Schilthuizen; Taxon Expeditions; individualCount: 1; sex: female; lifeStage: adult; preparations: card-mounted; disposition: in collection; **Taxon:** scientificName: Dermatohomoeus maliauensis; order: Coleoptera; family: Leiodidae; genus: Dermatohomoeus; specificEpithet: maliauensis; taxonRank: species; scientificNameAuthorship: Schilthuizen, Otani & Seip; **Location:** continent: Asia; island: Borneo; country: Malaysia; stateProvince: Sabah; municipality: Tongod; locality: Maliau Basin, near Studies Centre, along Seraya Trail, where stream enters the river; verbatimElevation: 260 m; verbatimCoordinateSystem: decimal degrees; decimalLatitude: 4.7389; decimalLongitude: 116.9696; **Event:** samplingProtocol: Winkler, litter from basis of trees; samplingEffort: c. 15 l of leaf litter; eventDate: 2017-09-27; fieldNumber: TxEx-MBSC1wb; **Record Level:** institutionID: Universiti Malaysia Sabah; collectionID: Institute for Tropical Biology and Conservation, BORNEENSIS; institutionCode: UMS; collectionCode: BORN; basisOfRecord: PreservedSpecimen
- **Type status:** Paratype. **Occurrence:** catalogNumber: BOR/COL/14093; recordedBy: I. Njunjić; M. Schilthuizen; Taxon Expeditions; individualCount: 1; sex: female; lifeStage: adult; preparations: card-mounted; disposition: in collection; **Taxon:** scientificName: Dermatohomoeus maliauensis; order: Coleoptera; family: Leiodidae; genus: Dermatohomoeus; specificEpithet: maliauensis; taxonRank: species; scientificNameAuthorship: Schilthuizen, Otani & Seip; **Location:** continent: Asia; island: Borneo; country: Malaysia; stateProvince: Sabah; municipality: Tongod; locality: Maliau Basin, near Studies Centre, along Seraya Trail, where stream enters the river; verbatimElevation: 260 m; verbatimCoordinateSystem: decimal degrees; decimalLatitude: 4.7389; decimalLongitude: 116.9696; **Event:** samplingProtocol: Winkler, litter from basis of trees; samplingEffort: c. 15 l of leaf litter; eventDate: 2017-09-27; fieldNumber: TxEx-MBSC1wb; **Record Level:** institutionID: Universiti Malaysia Sabah; collectionID: Institute for Tropical Biology and Conservation, BORNEENSIS; institutionCode: UMS; collectionCode: BORN; basisOfRecord: PreservedSpecimen

#### Description

Length of body c. 1.4 mm (Fig. 6). Oval, 1.45x longer than wide, greatest width at the shoulders. Pronotum 2.0x wider than the head. Winged. Reddish brown, with yellow legs and antennae. Eyes large. Antennae slender, antennomere VII 1.5x as long as wide. Pronotal posterior angle not drawn out, only a very faint convex curve next to the rounded angles. Elytra sparsely pubescent, with distinct punctuation, here and there arranged into longitudinal rows. All punctures on the elytra connected by microreticulation.

**Figure 9.**
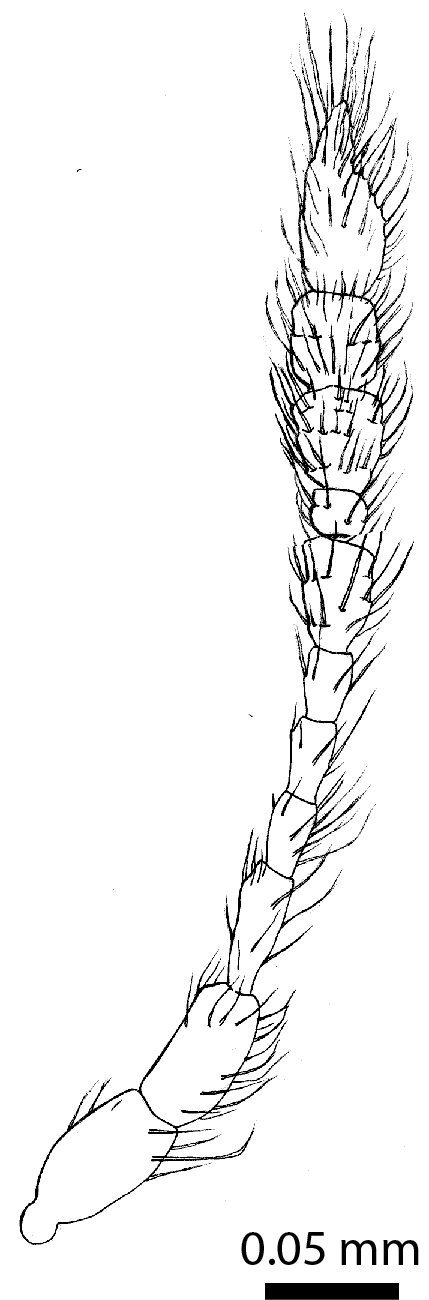
*Dermatohomoeus maliauensis* sp. n., male, antenna (drawn after electron micrograph) (paratype, FRCS)

**Head**: Eyes large, each with an estimated 40-50 ommatidia. Ratio of horizontal width of eye (measured in dorsal view and perpendicular to the longitudinal axis of the head) to distance between eyes: 1:4.4. A thin supraorbicular groove runs along the inner margin of the eyes and over the antennal insertion. Length of antennomere III 0.7 times the length of antennomere II. Antennomere IX slightly narrower than antennomere X. Antennomeres X and XI of equal width (Fig. 9). Punctuation (when viewed at 50x magnification) distinct, individual punctures spaced by 3-5 times their diameter (Fig. 7a). No microreticulation visible.

**Pronotum**: Broadest at the base. Pronotal posterior angle rounded, not drawn out, the pronotal basis near the posterior angle with only a very faint convex curve. Pronotum smooth, glossy, hairless, without any microreticulation but with very fine and sparse punctuation, punctures spaced at 3-5 times their diameter, nearly invisible at 50x magnification (Fig. 7b). A fine, continuous margin runs along the lateral and anterior margin.

**Scutellum**: Microsculptured as on pronotum.

**Elytra**: Broadest at the shoulders, roundly curved to apex. Elytra with distinct punctuation. Punctures separated by ca. 3x their own diameter, here and there arranged into longitudinal rows. All punctures connected by jagged horizontal striae that are spaced ca. 20 µm apart. Elytra with sparse hairs emerging from the punctures; these hairs are short, about as long as the width of antennomere III (Fig. 7c).

**Aedeagus**: Median lobe in dorsal view gradually narrowing towards the apex, terminally shaped into a broad, mushroom-shaped plate that is twice as wide as long (Fig. 8a). The apical angles of the ventral piece can be seen emerging on either side of this plate. Parameres reach the level of the basis of this plate. The dorsal surface of the terminal one-third of the median lobe is covered in coarse granules (only visible with scanning electron microscopy). In lateral view (Fig. 8b), the median lobe is basally strongly curved ventrad, but in the terminal one-third curved gently dorsad and flattened into the shape of a thin wedge.

**Spermatheca**: Not studied.

#### Diagnosis

As pointed out by Švec (2009), *Dermatohomoeus* species are often very similar externally and the male genitalia offer the only certain identification. The shape of the aedeagus separates *D. maliauensis* sp. n. from nearly all *Dermatohomoeus* species for which the male genitalia are known. One species with similar male genitalia is the widespread *D. portevini* (Champion, 1923). The size of the body and eyes, the microsculpture of the dorsum, and the shape of the pronotum indeed place the new species in the close vicinity of *D. portevini*. However, in *D. portevini* the median lobe of the aedeagus displays a bulge directly basal of the terminal plate (Daffner 1988), which is not the case in *D. maliauensis* sp. n. Also, the 7th antennomere is broader in *D. maliauensis.* A second species with a similar aedeagus is *D. bidentatus* Švec & Cooter 2015 from Yunnan, China which, however, is characterised by the two lateral teeth at the terminus of the median lobe; moreover, it is nearly twice as large (Švec and Cooter 2016). Another species that appears to possess similar external characteristics is *D. terrenus* (Hisamatsu 1985) from Korea and Japan. This species is thought to be parthenogenetic, as no males are known (Park and Ahn 2007). *D. terrenus* differs from *D. maliauensis* sp. n. in having an 11th antennomere that is distinctly broader than the 10th and by transverse microsculpture on the pronotum, which is absent in *D. maliauensis* sp. n.

#### Etymology

Named after Maliau Basin Conservation Area in Sabah, Malaysian Borneo. This 30-km-wide circular depression, covered with montane forest on poor soils and surrounded by steep sandstone cliffs, is known as "Sabah's Lost World". It is the focal area for the Borneo work of Taxon Expeditions. The species epithet was selected during a naming ceremony in Maliau Basin Studies Centre on 6 October 2017, in which expedition participants as well as a large number of field centre staff and porters took part. As far as the authors are aware, this is the third animal species named for this under-explored area (Disney 2016, Buhl 2009).

#### Distribution

Known only from two locations in the valley where the Maliau river flows out of Maliau Basin, located at 260 m elevation (Maliau Basin Studies Centre) and 670 m elevation (Ginseng Camp).

### Clavicornaltica

Scherer, 1974

Clavicornaltica Scherer, 1974; (Medvedev 1996, Scherer 1974, Konstantinov and Duckett 2005)
Clavicornaltica Clavicornaltica besucheti Scherer, 1974Scherer 1974

#### Diagnosis

Small (0.7-2.2 mm), convex flea beetles, with strongly developed jumping hind legs and characteristically clavate antennae. Frons is broad, antennal insertions widely separated. Antennae 11-segmented, clavate after the 3rd antennomere. The first three antennomeres are long and slender, the next three very small, the final five are enlarged and form a club. Pronotum with two setal pores, the anterior of which is placed behind the middle of the lateral margin. Metasternum with an anterior-pointing, broad processus. Posterior femora strongly dilated. Metatibia slender and with a long terminal spore and a row of smaller terminal setae on the lateral edge.

### Clavicornaltica sabahensis

Schilthuizen, Seip & Otani sp. n.

837800D6-5C3C-490F-9262-11603AA591BE

#### Materials

- **Type status:** Holotype. **Occurrence:** catalogNumber: BOR/COL/14089; recordedBy: Iva Njunjić; Menno Schilthuizen; Taxon Expeditions; sex: 1 female; lifeStage: adult; preparations: card-mounted; disposition: in collection; **Taxon:** scientificName: Clavicornaltica sabahensis; order: Coleoptera; family: Chrysomelidae; genus: Clavicornaltica; specificEpithet: sabahensis; taxonRank: species; scientificNameAuthorship: Schilthuizen, Seip & Otani; **Location:** continent: Asia; island: Borneo; country: Malaysia; stateProvince: Sabah; municipality: Tongod; verbatimLocality: Maliau Basin, near Studies Centre, along Seraya Trail, where stream enters the river; verbatimElevation: 260 m; verbatimCoordinateSystem: decimal degrees; decimalLatitude: 4.7389; decimalLongitude: 116.9696; **Event:** samplingProtocol: Winkler, litter from basis of trees; samplingEffort: 15 l of leaf litter; eventDate: 2017-09-27; habitat: lowland dipterocarp forest; fieldNumber: TxEx-MBSC1wb; **Record Level:** institutionID: Universiti Malaysia Sabah; collectionID: Institute for Tropical Biology and Conservation, BORNEENSIS; institutionCode: UMS; collectionCode: BORN; basisOfRecord: PreservedSpecimen

#### Description

Body dark reddish brown, small, oval and convex, ca. 0.75 mm long and ca. 0.58 mm wide (Fig. 10a). Eyes ca. 1/7 the width of the head. Antennae yellowish brown; clava long and moderately robust. Female wingless. Tibia and tarsus yellowish brown, femur dark brown and robust. Male unknown.

**Figure 11.**
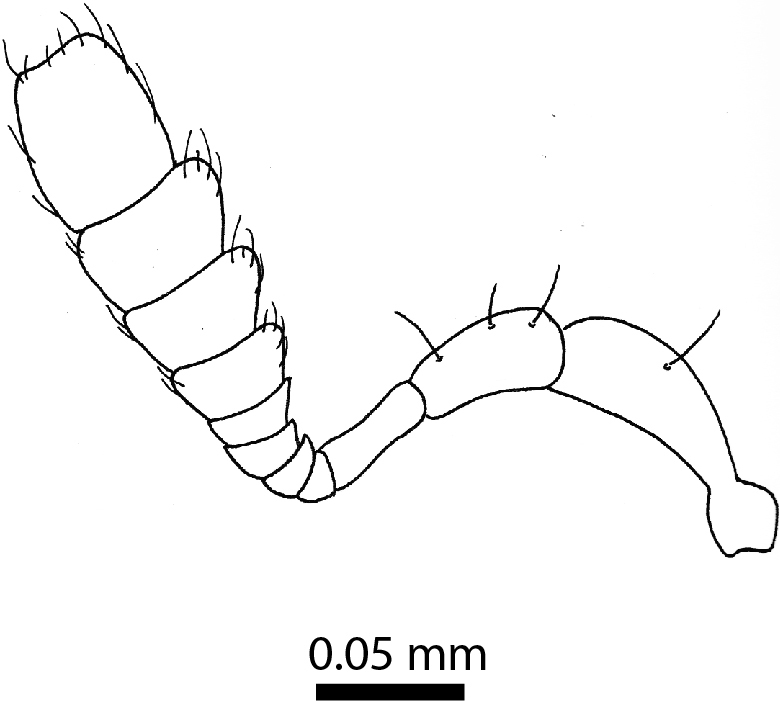
*Clavicornaltica sabahensis* sp. n., female, antenna (holotype, BOR/COL/14089).

**Head** (Fig. 10b): Rectangular, shallowly and sparsely punctate; vertex smooth; frontal tubercles present, frontal carina absent. Eyes ca. 1/7 the width of the head in dorsal view. Antennae: clava long and moderately robust (Fig. 11).

**Pronotum**: Lenticular in dorsal view, convex, four angles angular with a deep furrow along the length of the edge, which itself is somewhat angular in the middle with two deep seta-bearing pores: one at ¼ of the margin’s length, the other in the posterior angle. Punctuation covers the entire surface in an irregular pattern and is the same strength as the dorsal surface of the elytra.

**Hind wings**: Absent.

**Elytra**: Striae punctiform, punctures shallower dorsally, more deeply impressed laterally and becoming less visible towards the very apex. A deep but narrow punctuated groove runs along the entire margin continuing to the apex; apex itself slightly drawn out.

**Figure 12.**
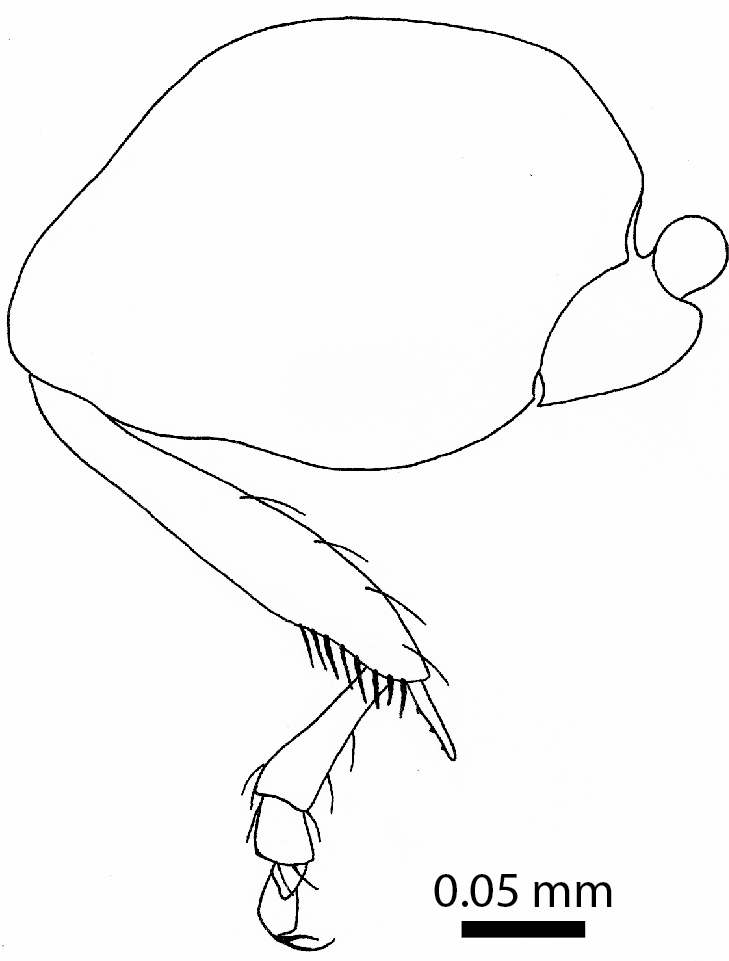
*Clavicornaltica sabahensis* sp. n., female, hind leg (holotype, BOR/COL/14089).

**Legs**: Metafemur robust, oval, covered in fine white setae. Metatibia bearing eight minute setae which cover the terminal one-quarter along the external edge and one long spine, slightly shorter than the first tarsomere, provided with three minute teeth (Fig. 12).

**Abdomen**: Carina on the first abdominal sternite sharp and narrow.

#### Diagnosis

Differential comparisons were made with all known species of the genus. *Clavicornaltica sabahensis* sp. n. differs from these in the following respects. *Clavicornaltica buechei* Medvedev, 2008 (Sulawesi): is larger (1.4 mm) and the ridge on the first abdominal segment is widened posteriorly (Medvedev 2008). *Clavicornaltica mizusawai* Suenaga & Yoshida, 2016 (Taiwan) has impunctate elytra and larger eyes (Suenaga and Yoshida 2016). *Clavicornaltica sakishimana* Suenaga & Yoshida, 2016 (Japan) has the carina on the first abdominal sternite widened caudally (Suenaga and Yoshida 2016). *Clavicornaltica pusilla* Scherer, 1974 and *C. loebli* Scherer, 1974 (Sri Lanka) have impunctate elytra (Scherer 1974). *C. besucheti* Scherer, 1974 (Sri Lanka) is larger, 1.5-2.2 mm (Scherer 1974). *Clavicornaltica malayana* Medvedev, 1996 (Malaysia) has the pronotum impunctate (Medvedev 1996). *Clavicornaltica iriana iriana* Medvedev, 1996 (New Guinea) is larger (1.2-1.4 mm), has larger eyes, only 4-5 rows of punctures on the elytra and these do not continue to the apex (Medvedev 1996). *Clavicornaltica iriana sarawacensis* Medvedev, 1996 (Borneo) is larger (1.2-1.4 mm) and the elytral punctures are only visible laterally (Medvedev 1996). *Clavicornaltica takizawai* Doeberl, 2009 (Nepal) is larger (1.45 mm) and its frons and vertex are densely punctuated (Döberl 2009). *Clavicornaltica mussardi* Scherer 1974 (Sri Lanka) is larger (1.3-1.5 mm) (Scherer 1974). *Clavicornaltica rileyi* Döberl, 2002 (India) is larger (1.5 mm) and its eyes are larger (Döberl 2002). *Clavicornaltica dali* Konstantinov & Duckett, 2005 (Yunnan): punctures on the head are stronger and those on the pronotum weaker than in *C. sabahensis* sp. n. (Konstantinov and Duckett 2005). *Clavicornaltica tarsalis* Medvedev, 1996 (Irian Jaya) is larger, 1.6 mm and the ridge on the first abdominal sternite is anteriorly broadly widened (Medvedev 1996). *Clavicornaltica australis* Konstantinov, 1996 (Queensland) lacks the long seta in the middle of the pronotal edge (Konstantinov and Duckett 2005). *Clavicornaltica fortepunctata* Scherer, 1974 (Sri Lanka): elytral punctuation fades abruptly before the apex (Scherer 1974). *Clavicornaltica trautneri* Medvedev, 1993 (Philippines) is much larger (2.1 mm) (Medvedev 1996). *Clavicornaltica takimotoi* Lesage, 1997 (Taiwan) is more globular and the punctures on the dorsum of the elytra are nearly invisible (Suenaga and Yoshida 2016). *Clavicornaltica philippinensis* Scherer, 1979 (Philippines) is larger (1.3 mm) (Medvedev 1996). *Clavicornaltica tamdao* Konstantinov & Duckett, 2005 (Vietnam): terminal setae on the metatibia are more numerous (12 in *C. tamdao* versus 8 in *C. sabahensis* sp. n.) and cover a larger section of the tibia (the terminal one third) (Konstantinov and Duckett 2005). *Clavicornaltica vietnamensis* Konstantinov & Duckett, 2005 (Vietnam): apex of the elytra is less extended (Konstantinov and Duckett 2005).

#### Etymology

Since this is the first species of *Clavicornaltica* found in Sabah, the specific epithet *sabahensis* ("inhabitant of Sabah") was chosen. This was one of several names suggested during a naming ceremony in Maliau Basin Studies Centre on 6 October 2017, in which expedition participants as well as a large number of field centre staff and porters took part.

#### Distribution

Known only from one location in the valley where the Maliau river flows out of Maliau Basin, at 260 m elevation (Maliau Basin Studies Centre).

#### Taxon discussion

Only a single female was at the authors' disposal. Nonetheless, the authors felt confident that this specimen represents an undescribed species. First of all, given their small size, apterism/brachypterism, and habitat (leaf litter in forests), it is unlikely that *Clavicornaltica* species have wide ranges (Konstantinov and Duckett 2005) and the only species known from Borneo and its immediate vicinity have external characters that do not match *C. sabahensis* sp. n. (see under Diagnosis). Moreover, *C. sabahensis* sp. n. carries a combination of traits that make it easily recognisable. Specifically, these are its extremely small size, highly vaulted shape, dark colouration, narrow ridge on the first abdominal sternite, angular, thick pronotal margin, and strong punctuation on the entire elytra.

## Discussion

While we believe that taxonomic work is best carried out in the context of large, genus-encompassing revisions by experts, we think that rapid taxon treatments of single species based on limited materials, such as we present here, have value. As digital techniques are becoming available that allow the aggregation of information from various sources, even small studies such as this one contribute to the knowledge of taxa. Provided that care is taken to (a) diagnose each species such that it can be recognised when found again and (b) avoid the introduction of junior synonyms, we think that even citizen scientists, if guided by properly trained taxonomists, can help close the large gap in the knowledge of the biodiversity of the world's invertebrates.

Moreover, the present results are the outcome of the first field trip of what is aimed to become a twice-yearly series of taxon expeditions to Maliau Basin. As our future work and publications will also focus on small leaf litter Coleoptera, we expect that knowledge of these and related, co-existing species will rapidly expand.

## Supplementary Material

XML Treatment for Colenisia

XML Treatment for Colenisia chungi

XML Treatment for Dermatohomoeus

XML Treatment for Dermatohomoeus maliauensis

XML Treatment for Clavicornaltica

XML Treatment for Clavicornaltica sabahensis

## Figures and Tables

**Figure 1.** Citizen scientists on the first Taxon Expedition in Malaysian Borneo performing leaf litter sieving. During this exercise, three new species of minute leaf litter beetles were discovered.

**Figure 2a.**
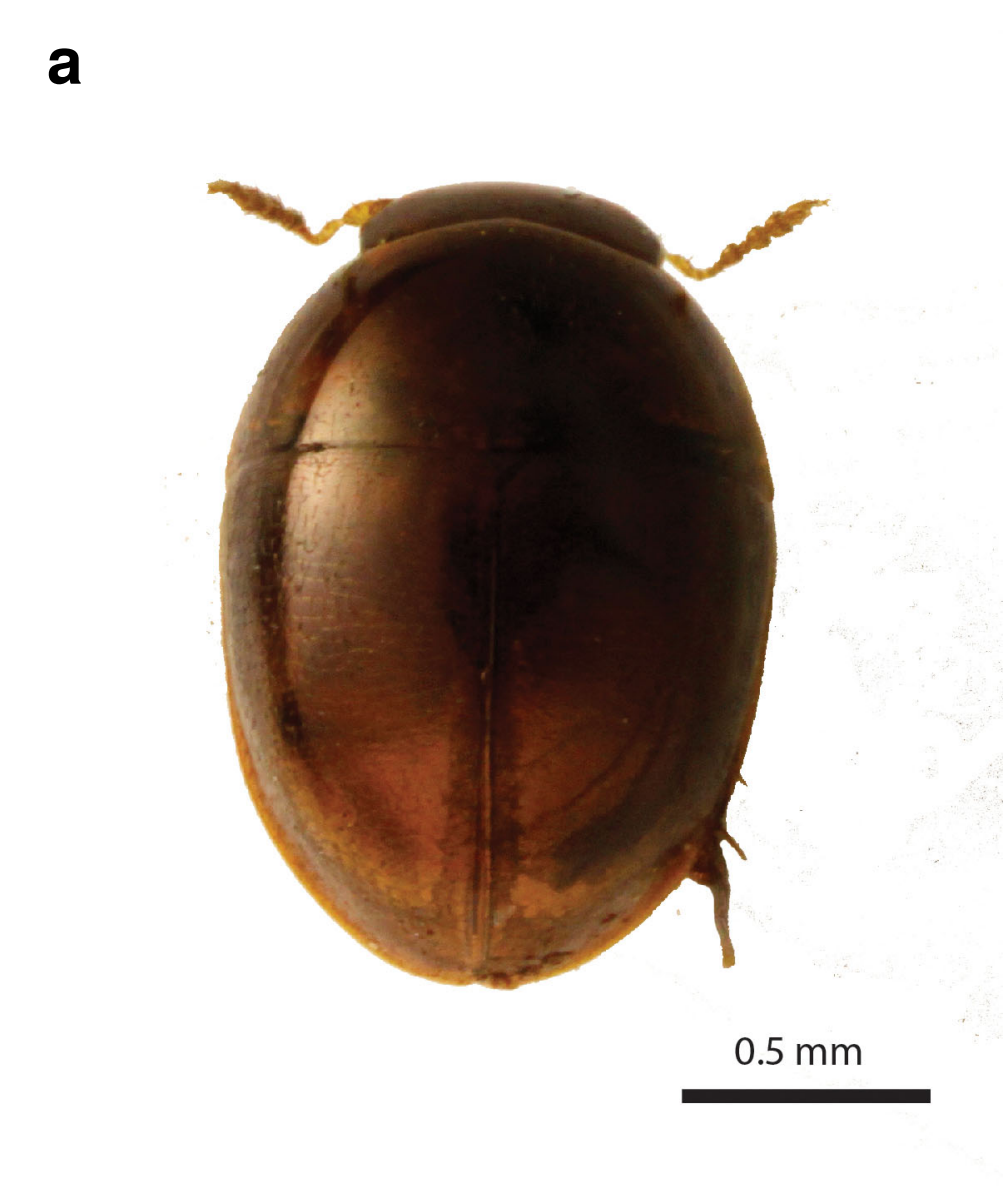
Female, dorsal view (paratype, BOR/COL/14091)

**Figure 2b.**
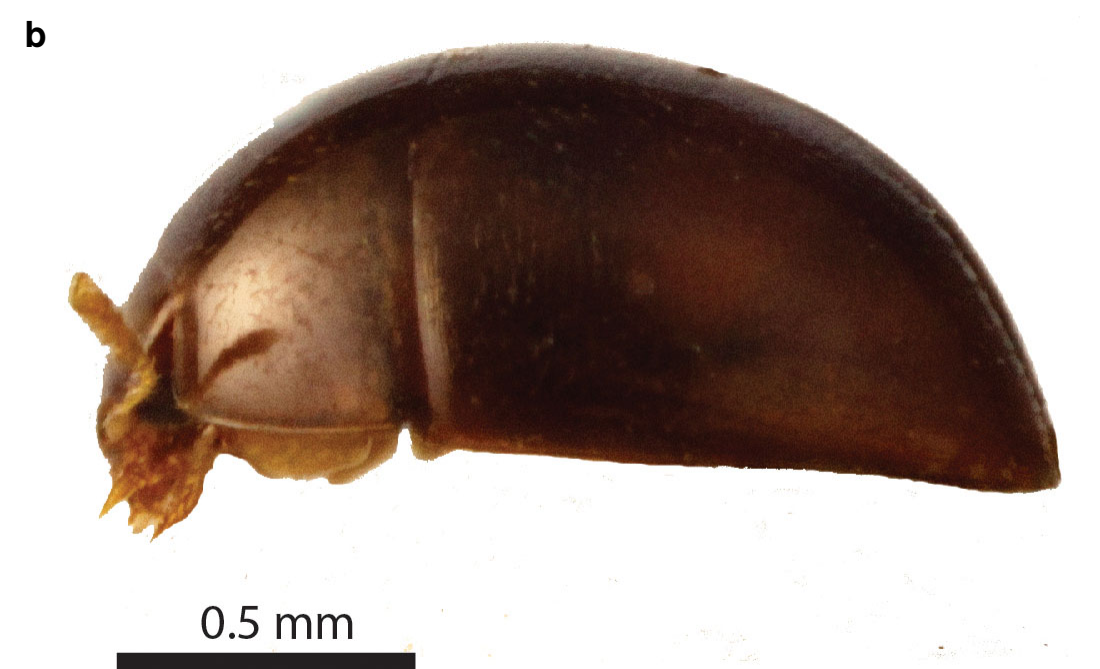
Female, lateral view (paratype, BOR/COL/14091)

**Figure 3a.**
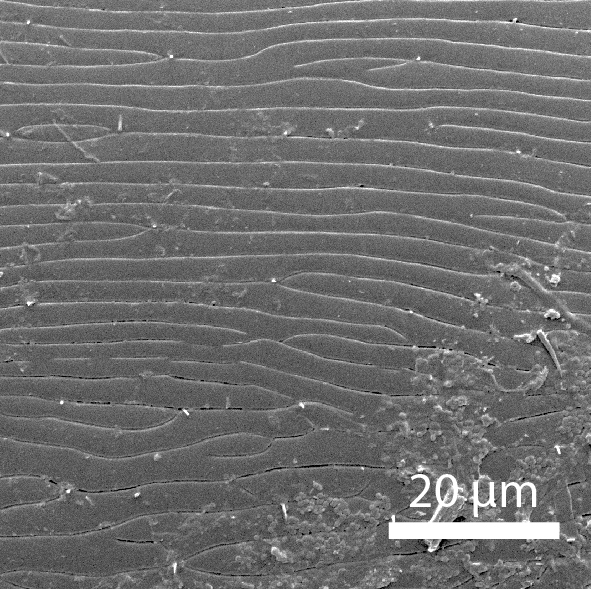
Male, head microsculpture (paratype, FRCS)

**Figure 3b.**
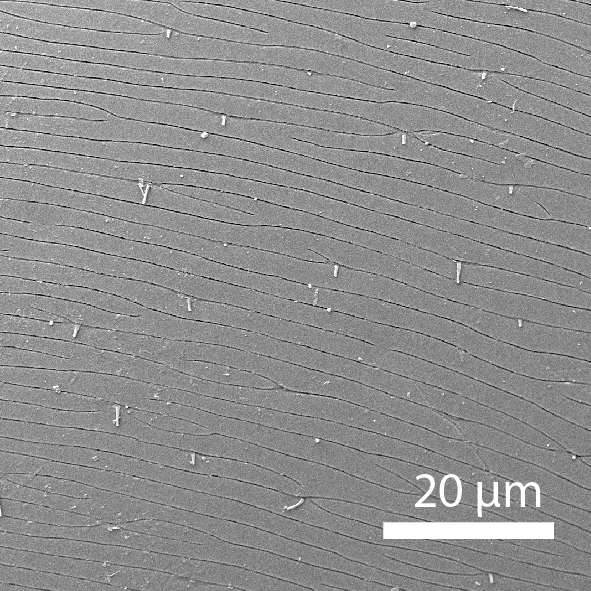
Male, pronotum microsculpture (paratype, FRCS)

**Figure 3c.**
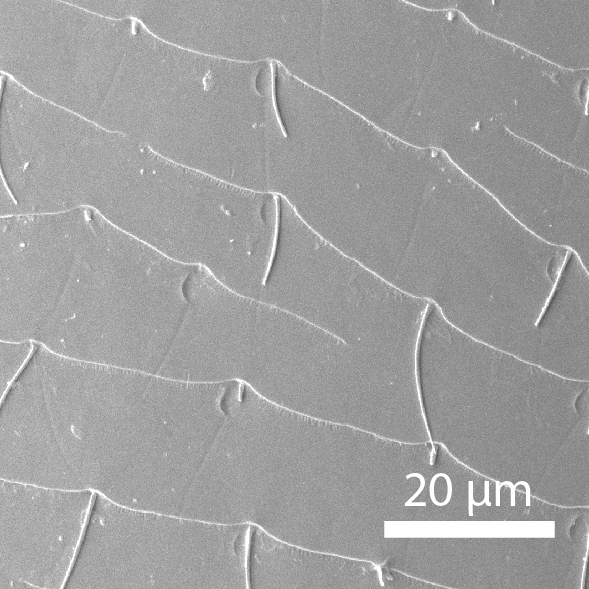
Male, elytra microsculpture (paratype, FRCS)

**Figure 4a.**
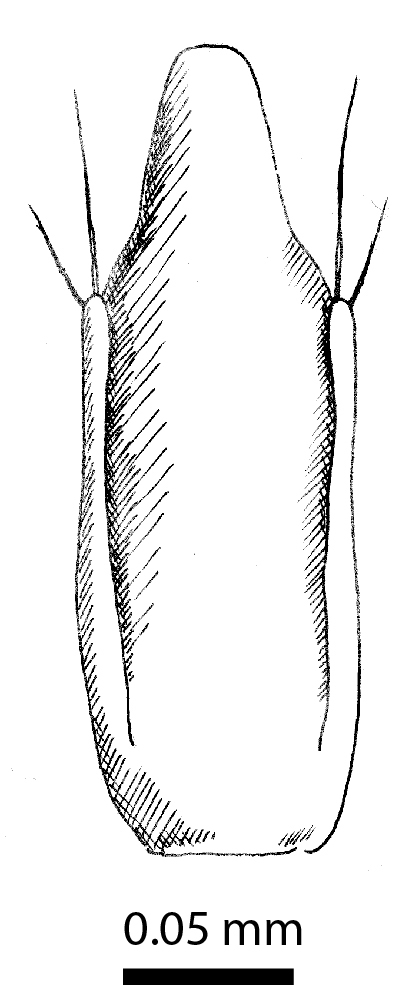
Male, aedeagus, dorsal view (drawn after electron micrograph) (paratype, FRCS)

**Figure 4b.**
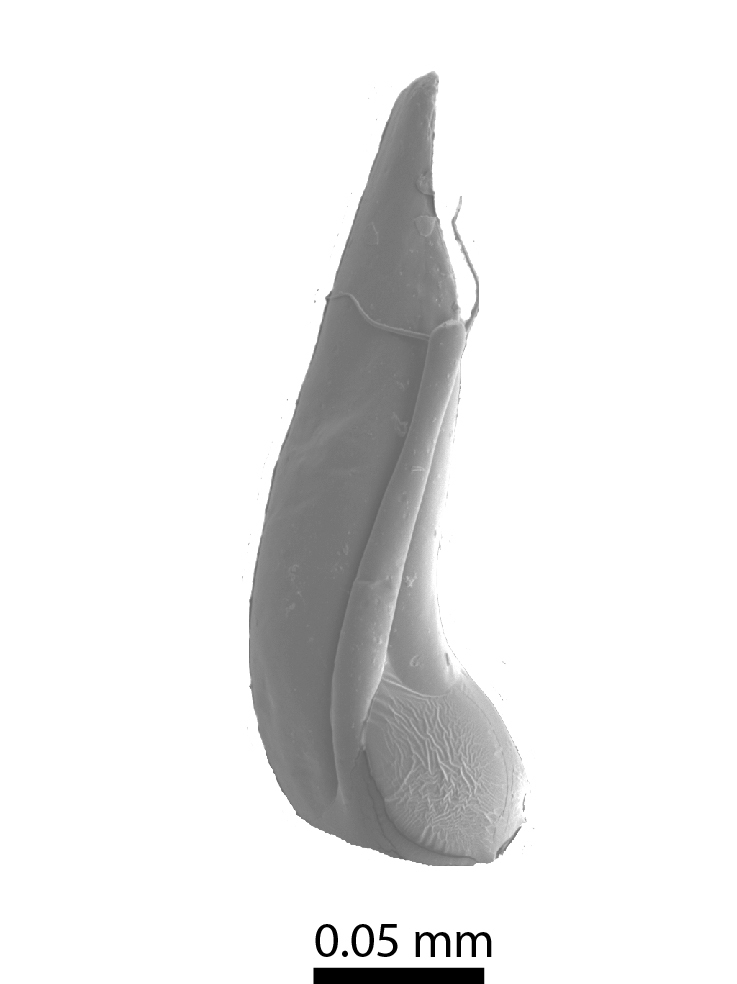
Male, aedeagus, lateral view (paratype, FRCS)

**Figure 5a.**
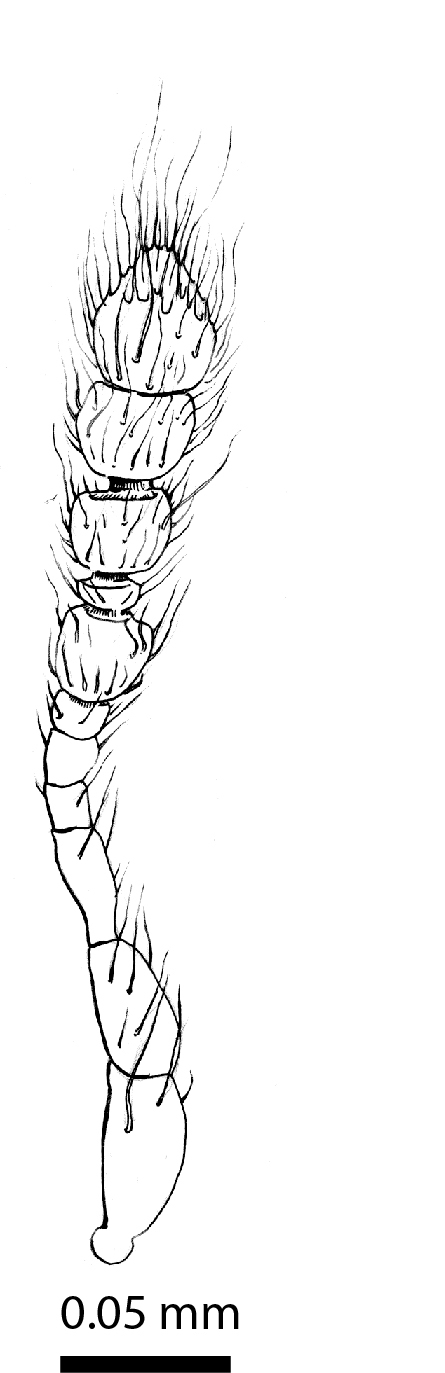
Male, antenna (drawn after electron micrograph) (paratype, FRCS).

**Figure 5b.**
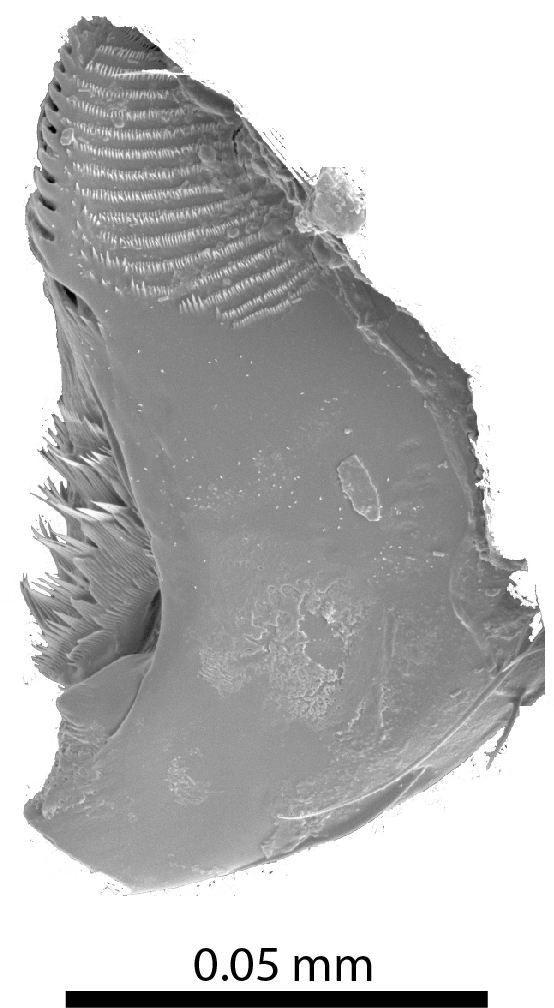
Male, left mandible, dorsal view (tip broken off) (paratype, FRCS)

**Figure 6a.**
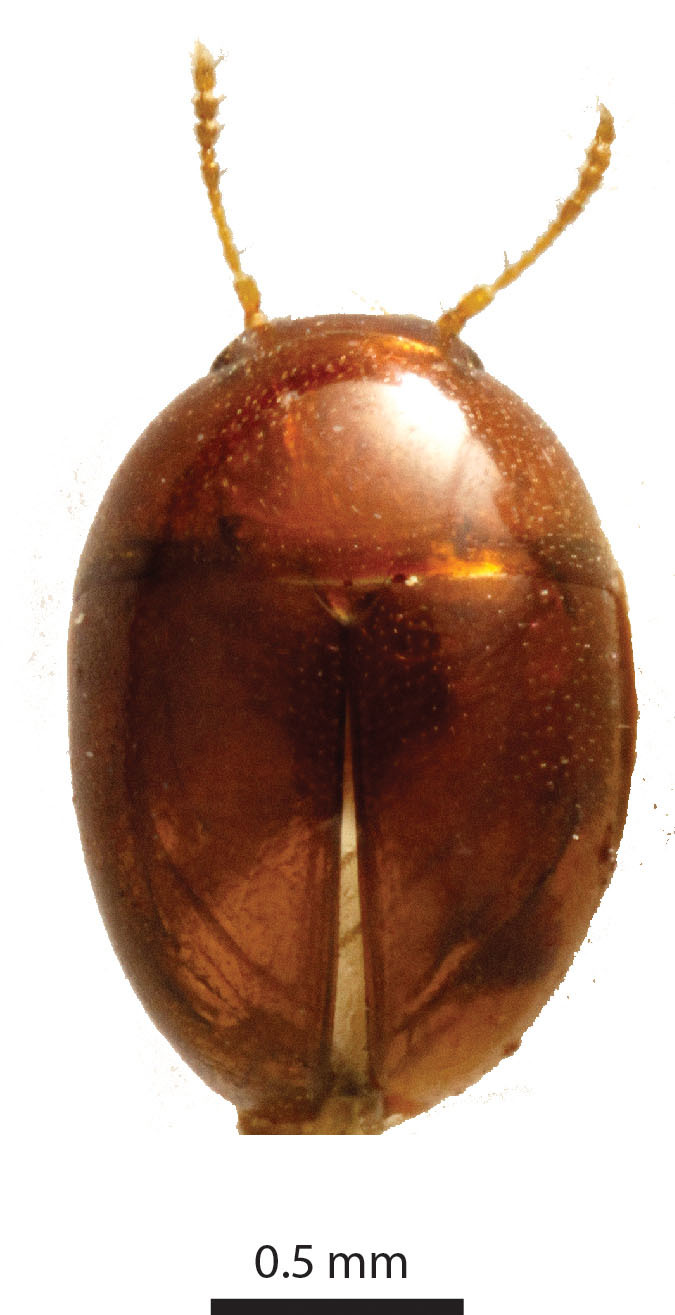
Female, dorsal view (paratype, BOR/COL/14093)

**Figure 6b.**
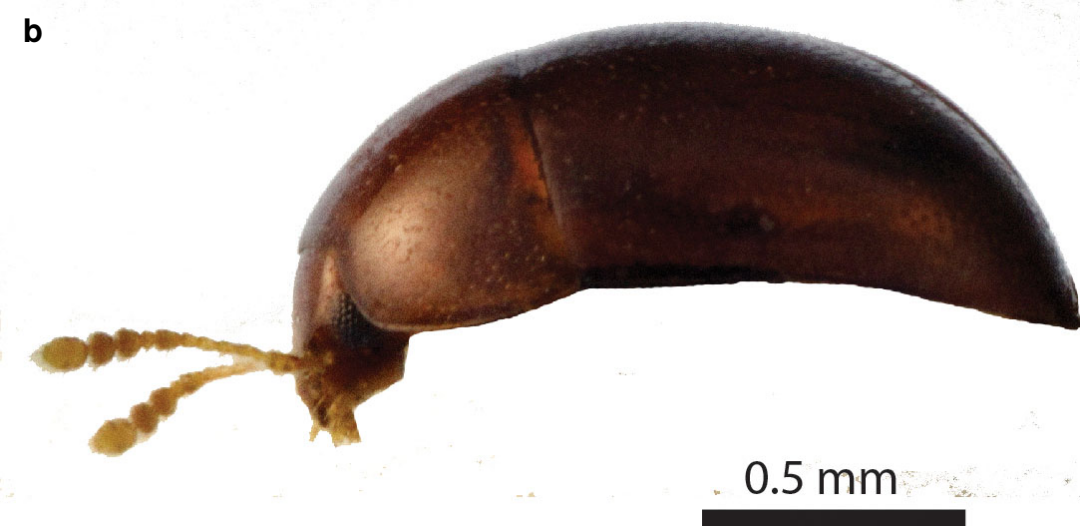
Female, lateral view (paratype, BOR/COL/14093)

**Figure 7a.**
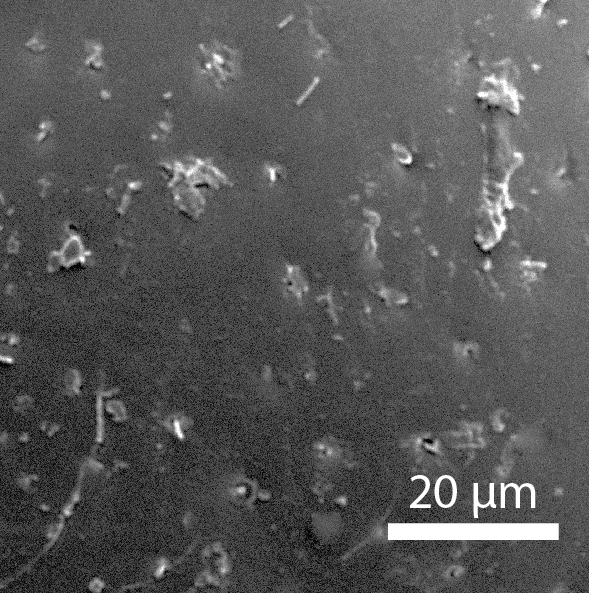
Male, head microsculpture (paratype, FRCS)

**Figure 7b.**
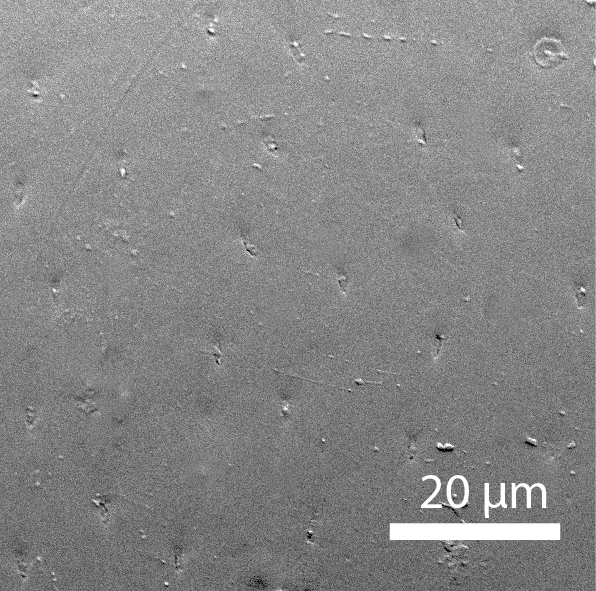
Male, pronotum microsculpture (paratype, FRCS)

**Figure 7c.**
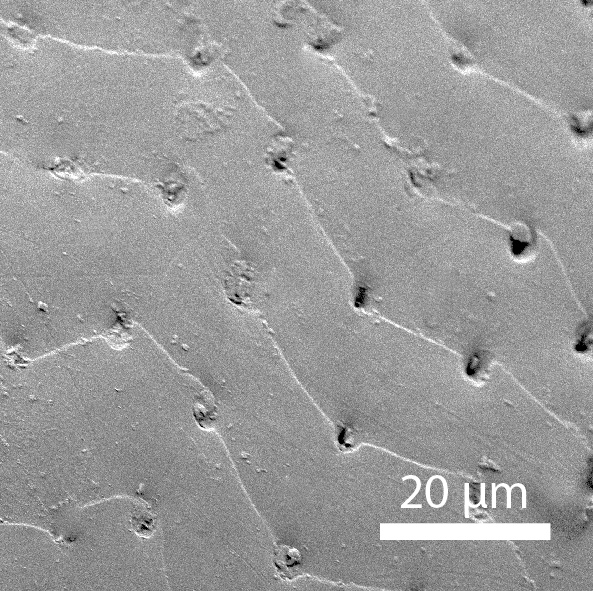
Male, elytra microsculpture (paratype, FRCS)

**Figure 8a.**
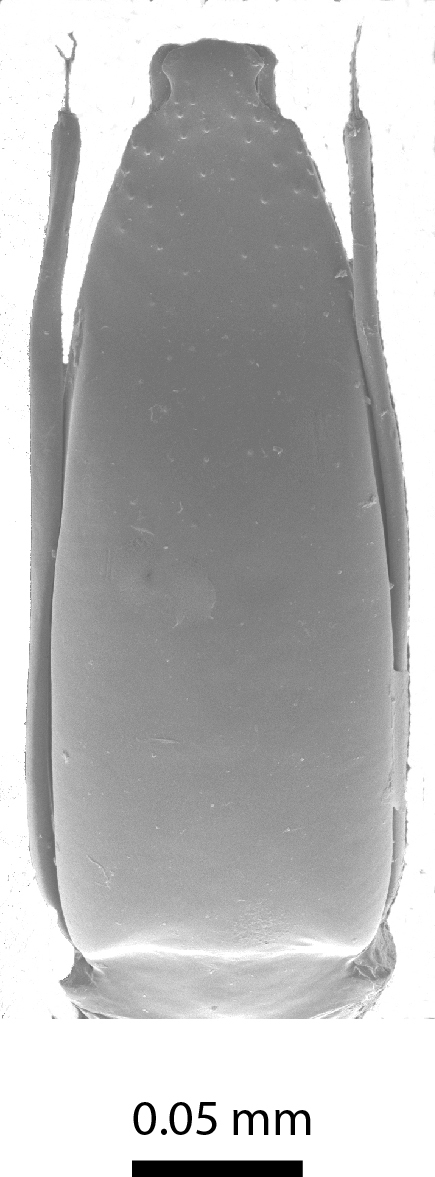
Male, aedeagus, dorsal view (paratype, FRCS)

**Figure 8b.**
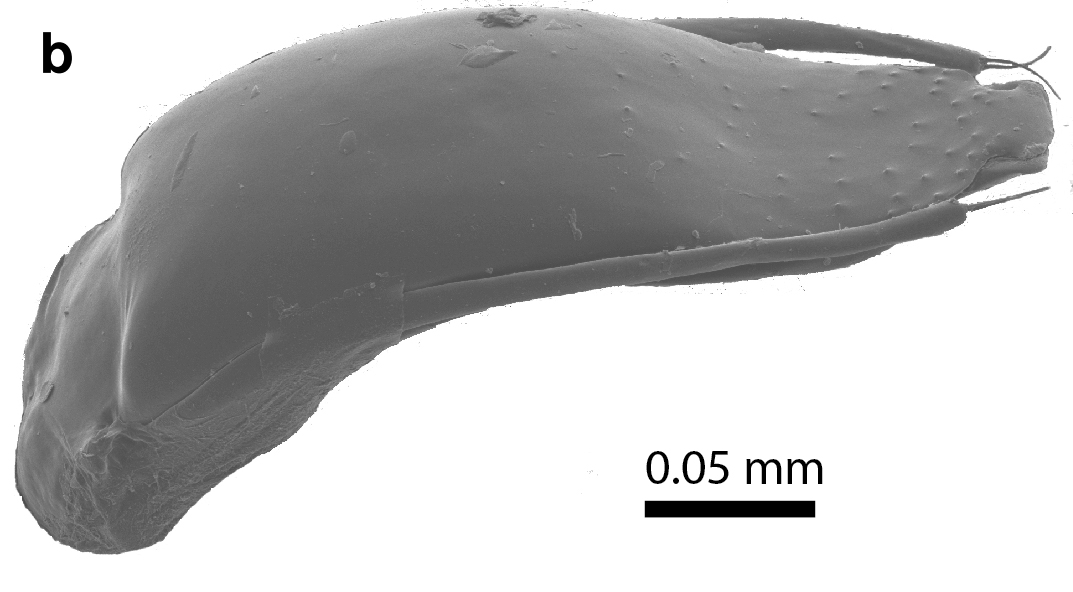
Male, aedeagus, lateral view (paratype, FRCS)

**Figure 10a.**
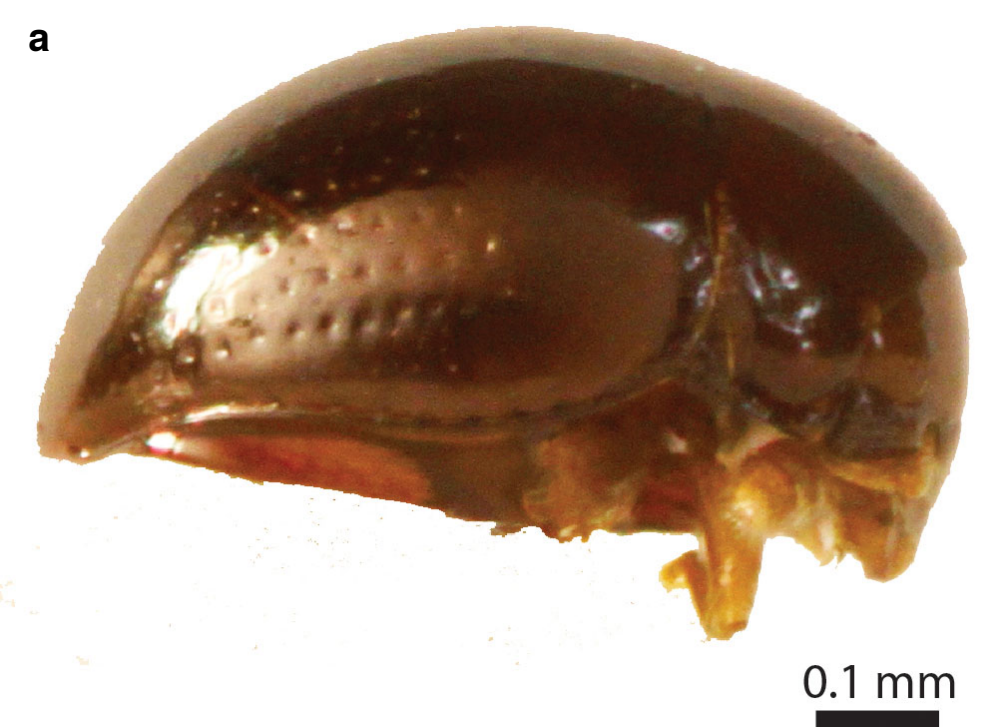
Habitus lateral view

**Figure 10b.**
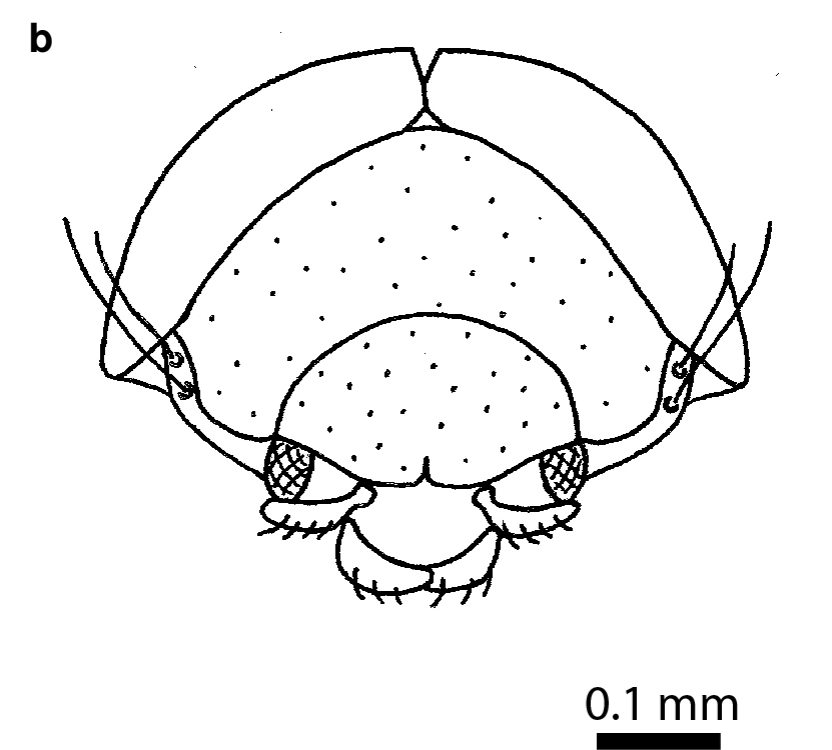
Habitus frontal view
